# Effects of mindfulness intervention on competition state anxiety in sprinters—a randomized controlled trial

**DOI:** 10.3389/fpsyg.2024.1418094

**Published:** 2024-10-31

**Authors:** Ming Ge Yu, Guang Bo Dou, Chen Gong

**Affiliations:** ^1^School of Physical Education, Northeast Electric Power University, Jilin, China; ^2^College of Kinesiology, Shenyang Sport University, Shenyang, China

**Keywords:** sprinters competition state anxiety, mindfulness-acceptance-insight-commitment (MAIC) training, cognitive state anxiety (CSA), state of self-confidence (SSC), somatic state anxiety (SSA)

## Abstract

**Objectives:**

With the rapid growth of China’s sprint program’s international competitiveness, the psychological problems of sprinters have become a common concern in sports training theory and practice. Hence, the study examined the impact of a 7-week Mindfulness training program on competition state anxiety in Chinese sprinters.

**Methods:**

Twenty-four sprinters (M_age_ = 22.46 ± 1.351) were selected in a 2 × 3 mixed design, with the group (mindfulness/control) as the between-subjects variable and test time (pre-test/mid-test/post-test) as the within-subjects variable. The dependent variables corresponded to the mindfulness score and competition state anxiety score. One 60-min session was conducted once a week for 7 weeks, and the control group did not undergo any psychological training. The mindfulness group received mindfulness training, and the control group received regular psychological guidance. The subjects filled in the Five Facet Mindfulness Questionnaire (FFMQ) and the Competition State Anxiety Scale at baseline, followed by additional assessments 3 weeks and 7 weeks later.

**Results:**

(1) In terms of competition state anxiety, there were no significant differences in the pre-test between the mindfulness group and the control group. There were significant differences in sprinters’ competition state anxiety after mindfulness intervention in terms of time, group, and the interaction between time and groups (*p* = 0.03, 0.004, and 0.009). (2) In terms of the mindfulness level, the difference between the mindfulness group and the control group was not significant in the pre-test. The sprinters’ mindfulness level was significant in the interaction between groups and that between groups and time after mindfulness intervention (*p* = 0.027 and 0.028).

**Conclusion:**

Mindfulness training alleviated sprinters’ competition anxiety by reducing sprinters’ somatic state anxiety (SSA) and cognitive state anxiety (CSA), as well as improving state self-efficacy. The results provide guidance and references for Chinese sprinters’ psychological problems.

## 1 Introduction

Competition anxiety has been considered to be one of the most studied fields in sports psychology (Ong and Chua, 2021; Daley et al., 2024; Boughattas et al., 2022). It is defined as a response to the characteristics and/or similar states of stressful exercise-related situations. Individuals believe that this response is potentially stressful, which leads to a series of cognitive assessments, behavioral responses, and/or physiological arousal (Ford et al., 2017).

Past meta-analysis has proven the influence of competitive status anxiety on sports performance (Woodman and Hardy, 2003). CSA has a significantly negative impact on sports performance. In addition, high anxiety scenarios cause athletes to perform excessive error monitoring (Masaki et al., 2017) and reduce anticipation timing performance (Duncan et al., 2016). Competition anxiety increases the risk of sports injury for sprinters (Ford et al., 2017). Competitive status anxiety is a risk factor for skeletal muscle injury in sprinters (Cagle et al., 2017).

Chinese sprinters have demonstrated remarkable progress in their performance through scientific training methods in recent years, which resulted in a substantial and comprehensive advancement during the Tokyo Olympic cycle and a historic breakthrough (Xue Feng, 2022). Su Bingtian, clocking in at 9.83 s, established a new Asian record and secured his place in the men’s 100-m semifinals at the Tokyo Olympics, which makes him the first Chinese athlete to advance to the final of this event (Wang, 2023). Ge Manqi and Xie Zhenye advanced to the women’s 100 m and men’s 200 m semifinals at 11.20 and 20.34 s, respectively. Simultaneously, Su Bingtian, along with Tang Xingqiang, Xie Zhenye, and Wu Zhiqiang, secured the bronze medal in the men’s 100-m relay event, which achieved the highest level of participation by the Chinese Olympic delegation. Wu Yanni and Lin Yuwei, both accomplished athletes in women’s 100-m hurdles, qualified for the 2024 Paris Olympics (Hongren and YaPing, 2022).

An increasing number of exceptional sprinters with remarkable achievements and distinctive characteristics are being discovered in the era of new media, which attracts attention and admiration from online users. Consequently, they have emerged as prominent Internet celebrity athletes. However, athletes’ popularity on the Internet can overly increase athletes’ exposure, which counters athletes’ confident and optimistic sports mentality (Yue Hui and Xiangyang, 2023). The web celebrity status of sports stars means that they are under the panoramic surveillance of the network society and the expectations of the public and coaches (Kim et al., 2019). Web celebrity athletes with poorer mental health need to be acknowledged by public opinion, as they often experience psychological pressure stemming from the desire to win and the fear of losing (Xiao Li et al., 2022). Reardon et al. (2024) shows that excessive psychological stress in athletes can cause cognitive and somatic anxiety, negatively affecting the quality of their training and competition.

Improving sports performance has always been a basic goal for athletes to excel in their respective sports. Regardless of the time and effort spent, athletes continuously try to improve their skills and athletic level. According to Behroz’s research, many athletes in professional teams are committed to training throughout the year and improving their sports performance to obtain bonuses or reach a professional level. Therefore, they win the high hopes of parents and coaches (Khodayari et al., 2011). Unfortunately, high hopes increase the pressure on athletes, which usually causes athletes’ competition anxiety. When anxiety is not guided, athletes lose control, which decreases athletic performance (Cox et al., 2003).

Regarding the athlete cultivation system in China, the predominant focus on training and competitions results in an environment where many athletes are primarily centered around sports activities. Consequently, this emphasis restricts the development of a well-rounded social personality and a scientific perspective on life (Dennis et al., 2024). When the technical level reaches a certain height, athletes have ambiguous developmental goals, poor comprehension of high-level events, and poor adaptability due to the shortcomings of early emotional intelligence and personality shaping. The plateau phenomenon (Miller et al., 2024) of athletic ability further restricts the development of their athletic ability to a higher level. Targeted interventions should be implemented in their psychological wellbeing to facilitate the advanced development of athletes.

Concerning types of sports, individual sport athletes have higher cognitive anxiety scores and lower self-efficacy scores than group sport athletes (Pluhar et al., 2019). Individuals participating in individual sports who rely on distance mobilizers exhibit a higher susceptibility to competition anxiety than athletes engaged in team sports (Hossein et al., 2016).

A range of psychological interventions have been used in previous studies to assist athletes in managing competition-related anxiety. A variety of psychological interventions have been adopted to help athletes cope with anxiety in the competitive state. Psychological health problems are reduced (Schinke et al., 2021) to ensure that they do not affect athletes’ training and competitions. Previous research has focused on utilizing various psychological techniques to aid athletes in coping with competition-induced anxiety [e.g., mental training (Fekih et al., 2021), self-talk training (Walter et al., 2019), and progressive relaxation training (Hussein et al., 2019)]. They are called psychological skill training (PST). Additional psychological interventions such as mindfulness training (Mehrsafar et al., 2019) and biofeedback training (Pusenjak et al., 2015) have gained increasing attention. Mindfulness intervention and PST are commonly used by most sports teams to deal with competition anxiety. Compared to mindfulness training, control-based PST emphasizes control to obtain optimal internal experience. However, the effect is passable. In contrast, mindfulness training that does not emphasize control and optimal internal experience allows athletes to focus more freely on current tasks (Gardner and Moore, 2004; Moore, 2009). Therefore, mindfulness training is used to intervene in the competition state anxiety of sprinters in this study.

Mindfulness has been described as “awareness that emerges through paying attention on purpose, in the present moment, and non-judgmentally to the unfolding of experience moment by moment” (Kabat-Zinn, 2019). Mental training based on mindfulness and acceptance has generated a great deal of interest and concern among researchers and practitioners in sports psychology, particularly within the context of the third wave of cognitive therapy (Bühlmayer et al., 2017). Mindfulness in this psychological training is derived from the Eastern Buddhism of Nei Kuan and Zen, as well as Western psychology. When combined with contemporary Western culture, it pioneers the integration of ancient Eastern wisdom into clinical psychotherapy and counseling, leading to the development of a range of mindfulness and acceptance-based mental training methods (Gang Yan et al., 2014).

Mindfulness was first applied by Kabat-Zinn in the context of athletes’ daily training and Olympic preparation in sports (Demarzo, 2014). Mindfulness training and acceptance have attracted attention in sports due to their application and development in clinical and counseling psychology, addressing limitations in traditional psychological training. Research in sports suggests that this particular type of attention may be beneficial (Bühlmayer et al., 2017). Fortunately, mindfulness training methods have been developed specifically for athletes. The mainstream training methods with more systematic theories and wider application include mindfulness-acceptance-commitment (MAC) (Gardner and Moore, 2017), mindfulness sports performance enhancement (MSPE) (Hut et al., 2021), mindfulness-acceptance-based-interventions (MABIs) (Minkler et al., 2024), and mindfulness-acceptance-insight-commitment (MAIC) (Su et al., 2019).

In contrast to traditional psychological skills, the mindfulness training approach focuses on mind–body experiences (e.g., thoughts, physical sensations, and emotions) (Mohebi et al., 2021) rather than controlling or altering these experiences (Röthlin et al., 2020). The mainstream approaches to mindfulness training in sports have been widely utilized. They enhance athletic performance (Chang et al., 2023) by improving concentration (Gao and Zhang, 2023), flow state (Augustus et al., 2023), and confidence (Oguntuase and Sun, 2022) as well as reducing burnout (Zhang et al., 2023) and exercise anxiety (Blanck et al., 2018).

Positive mindfulness is negatively correlated with cognitive and somatic anxiety in cross-sectional designs, suggesting that positive mindfulness may reduce competition anxiety. Improved mindfulness positively affects performance-related factors such as emotional processing and attention control (Röthlin et al., 2020). MAIC can enhance athletes’ attentional control, emotional processing, and resilience to failure (Röthlin et al., 2020), contributing to their overall psychological wellbeing (Evers et al., 2021).

The psychological problems of sprinters have become a common concern in sports training theory and practice with the rapid growth of the international competitiveness of China’s sprint program. The study examined the effects of 7 weeks of systematic mindfulness training on the competition anxiety of sprinters, providing guidance and implications for Chinese sprinters.

## 2 Methods

### 2.1 Participants

The study recruited 24 athletes from sprinters (M_age_ = 22.46 ± 1.351; 14 males and 10 females; 11 people with training years of less than 6 years and 13 people with training years of more than 6 years) through the Jilin Province Sports Association. G * Power (Faul et al., 2009) (power = 0.90, *α* = 0.05, and effect size *f* = 0.37) was used for a previous efficacy analysis to estimate the minimum sample size. According to the calculation results, at least 18 sample sizes were required to test the variance-analysis interaction terms of 3 (time) × 2 (condition) repeated measurement. The Competitive State Anxiety Inventory-2 (CSAI-2) was issued to sprinters who met the standards through the Jilin Province Sports Association, and 232 sprinters were assessed for competitive status anxiety. Sorted using the anxiety scores, sprinters who met the criteria were invited to participate in the study. The inclusion criteria were as follows: participants had to be at least 18 years old; have no prior experience with mindfulness studies or participation in similar experimental studies or psychological interventions; and possess a mean competition anxiety score of 3.5 (derived from the average score of 232 sprinters, with competition state anxiety was calculated, and the average score of 25% of sprinters with higher competition state anxiety was 3.5). Additionally, participants must not have a history of psychiatric disorders; they should be capable of engaging in regular training sessions and competitions; they should be eligible to compete in the 100-m dash in the 21st National Collegiate Athletics Championships. Eligible athletes were randomly assigned to the mindfulness group and control group based on a computer-generated list of random times. Initially, 25 participants completed the pre-intervention survey with one excluded from the study. There were 24 final valid subjects, with 12 in the mindfulness group and 12 in the control group (Table 1). All athletes ultimately included in the analysis participated in all mindfulness sessions.

**Table 1:** Participants demographics.

Characteristics	MAIC (*n* = 12)	Control (*n* = 12)	*P*
Age (M ± SD)	22.58 ± 1.311	22.33 ± 1.435	
Gender (%)			1.000
Females	41.7%	41.7%	
Males	58.3%	58.3%	
Training age (%)			0.698
5 years or less	41.7%	58.3%	
6 years and above	50.0%	50.0%	

MAIC: mindfulness training group. Control: control group. M: Mean. SD: standard deviation.

### 2.2 Procedure

Randomization was used to assign subjects to the mindfulness (intervention) group (*n* = 12) and the control group (*n* = 12). Athletes received interventions in the conference room of the university gymnasium every Saturday morning over 7 weeks, with each session lasting 60 min and occurring once weekly. The mindfulness group participated in a 7-week mindfulness intervention based on the mindfulness training content, while the control group did not receive guided exercises for mindfulness intervention. They were instructed to sit comfortably, relax, and allow their thoughts to wander freely, such as recalling daily life or imagining the future. A single-blind design (Kimberly MacLin, 2023) was adopted to control unrelated variables. The purpose and process arrangements of the test were kept confidential from the athletes. All participants were assessed in the pre-intervention stage (T0), the mid-intervention stage (T3), and the post-intervention stage (T7) (Hao, 2021). The Five Factors of Mindfulness and Competitive State Anxiety Scales were distributed to participants. The mindfulness intervention was facilitated by a sports psychologist who held a certification in mindfulness-based practices. All subjects signed the consent forms and had the right to withdraw at any time during the experiment. They were informed that they would receive a reward based on the completion of the tests.

### 2.3 Study design

A 2 * 3 mixed experimental design was used. The effects of mindfulness training on mindfulness levels and competition anxiety in sprinters were examined. The group (mindfulness/control) was the between-subjects variable. Test time (T0 pre-test/T3 mid-test/T7 post-test) was the within-subjects variable. The dependent variables corresponded to the levels of mindfulness and competition anxiety. Athletes participated in the intervention in the conference room of the university gymnasium every Saturday morning. Each athlete was asked to perform 15 min of breathing exercises at home every day for 7 weeks after training. The insights gained from their pre-session practice before each mindfulness session were analyzed.

### 2.4 Mindfulness intervention

The intervention consisted of a 7-week MAIC program. The content was based on the *Mindfulness Training Manual for Athletes* by Si et al. and developed from the interviews of professional coaches in track and field. Mindfulness interventions were conducted by a master of sports psychology who was certified in mindfulness intervention. Specifics included the following aspects: (1) Preparation for mindfulness training included introducing the theory, facts, and basic methods of mindfulness training and helping athletes form a preliminary impression of the activity and arouse interest in participating. (2) Mindfulness included experiencing mindfulness through practice and guiding athletes to understand mindfulness in practice. (3) De-centering included helping athletes gradually shift from preoccupation to task attention through practice. (4) Acceptance included the mindfulness raisin exercise and mindfulness drinking practice, as well as experiencing and accepting all inner emotions and thoughts. (5) Values and awareness included helping athletes clarify the current direction of behaviors. (6) Commitment included transforming values into specific behaviors, helping athletes realize values, overcoming obstacles in the realization process, applying mindfulness, decentration, and receptive skills, and adhering to effective behaviors for values. (7) Integration included reviewing and synthesizing the skills learned in the first 6 classes so that athletes could overall understand mindfulness training. Table 2 shows the specific contents.

**Table 2:** Mindfulness-acceptance-insight-commitment (MAIC) training content.

Training topics	Training content and objectives
Mindfulness preparation	Introduce the theory, facts, and basic methods of mindfulness training; help athletes form a preliminary impression of the activity and arouse interest in participating.
Mindfulness	Body scanning exercises; assist athletes to fully understand their status, maintain objectivity, do not judge and react, and focus on the current situation.
Egocentrism reduction	Explain the concept of self-centeredness: guide athletes to gradually shift from self-preoccupation to task attention; perform mindfulness imagery exercise and mindful eating of grapes.
Acceptance	Mindfulness raisin exercise and mindfulness drinking practice; do not judge, do not react to inner experiences, and accept their existence as they are. Do not evaluate objective things with personal subjective intentions; instead, recognize and accept them as they are.
Value and consciousness	Mindfulness breathing and consciousness; focus on values and awareness; guide athletes to discover the meaning of their current behavior and the direction and motivation of thinking behavior, as well as to take responsibility for their behavior.
Commitment	The extension of values and consciousness: Values serve as the guiding compass and motivational impetus for athletes’ behavior, while concentration affords them the means to allocate their time effectively. As athletes refine their state of consciousness, they become more adept at resolutely committing to and engaging in behaviors that resonate with their fundamental values.
Comprehensive review and consolidation	A comprehensive review and synthesis of the training provided in mindfulness intervention is provided, along with an integrated direction and guide for mindfulness practice and application after the mindfulness training course.

### 2.5 Measures

#### 2.5.1 FFMQ

The FFMQ (Baer et al., 2006), translated and revised by Deng et al. (2011), includes 39 items. Five aspects are used to assess mindfulness: observing, describing, acting with awareness, non-judging of inner experience, and non-reacting to inner experience. The FFMQ has acceptable psychometric properties. Its subscales measure individual characteristics or traits (Kabat-Zinn, 2003). The scale utilizes a 6-point Likert scale format from 1 (not at all) to 6 (exactly). Higher scores reflect higher levels of mindfulness.

The FFMQ was used to calculate the subscale scores. The items were reverse-coded. The subscale scores were calculated by summing item scores. SPSS.27 was used for the reliability analysis of the FFMQ. The internal consistency of the current sample was 0.825 (Cronbach’s *α* at baseline). Bartlett’s test of sphericity, based on chi-square, showed a result of 1,945.061, with 136 degrees of freedom (*p* < 0.001). Cronbach’s *α* for the subscales demonstrated good reliability: observing (0.783), describing (0.623), acting with awareness (0.873), non-judging of inner experience (0.799), and non-reacting to inner experience (0.642).

#### 2.5.2 CSAI-2

The CSAI-2 (Cox et al., 2003) includes 27 items and 3 subscales: cognitive, somatic, and self-confidence anxiety. The inventory uses a 6-point Likert scale from 1 (not at all) to 6 (completely). Higher total scores are associated with higher levels of anxiety. There are 14 reverse-coded questions. The subscale scores were calculated by adding up the item scores. SPSS.27 was used for the reliability analysis of the CSAI-2. The internal consistency of the current sample was 0.899 (Cronbach’s α at baseline). Bartlett’s test of sphericity, based on chi-square, showed a result of 1,945.061, with 136 degrees of freedom (*p* < 0.001). Cronbach’s α for the scale and its subscales ranged from 0.694 to 0.900, indicating high reliability.

### 2.6 Data analyses

The normality test (Shapiro–Wilk test) was performed before the statistical analysis. The independent t-test for normally distributed variables was used to compare the baseline variables of the two groups. General linear models were used to examine the impact of a 7-week mindfulness training program on competitive state anxiety by analyzing pre-intervention, mid-intervention, and post-intervention data. The between-group effects of the mindfulness training and control groups were further tested when the difference between the two groups was statistically significant. ANOVA and Bonferroni correction were used for multiple comparisons. *η^2^*_p_ of the interaction effects of time and group was reported for two-by-two comparisons. The cutoff value of *η^2^*_p_ was as follows: *η^2^*_p_ < 0.019 represented a minute effect size; 0.02 < *η^2^_p_* < 0.059 represented a small effect size; 0.06 < *η^2^*_p_ < 0.139 represented a medium effect size; *η^2^*_p_ > 0.14 represented a large effect size (Cohen, 1988). All data were analyzed using SPSS 27.0. A *p*-value of ≤0.05 indicated statistical significance.

## 3 Results

### 3.1 FFMQ and CSAI-2 before mindfulness intervention

There were no statistically significant differences between the distribution of the mindfulness group and the control group in terms of gender and years of training (Table 1). There were no statistically significant differences between the two groups in terms of somatic, cognitive, and self-confidence anxiety, as well as in the dimensions of observing, describing, acting with awareness, non-judging of inner experience, and non-reacting to inner experience (Table 3).

**Table 3:** Descriptive statistics for study variables including t-test comparisons between groups.

	MAIC	Control	*T*	*P*
	M ± SD	M ± SD		
CSA	27.00 ± 7.97	30.58 ± 6.35	−1.219	0.236
SSA	27.00 ± 10.20	26.83 ± 7.74	0.045	0.685
SSC	38.92 ± 8.66	35.33 ± 7.16	1.104	0.281
CSAI-2	92.92 ± 14.74	90.67 ± 14.03	0.383	0.469
Observe	29.83 ± 6.46	28.58 ± 7.21	0.447	0.659
Describe	28.08 ± 6.69	26.08 ± 4.38	0.433	0.669
Non-judge	26.58 ± 4.32	26.58 ± 4.32	0.441	0.663
Non-react	25.25 ± 3.06	25.25 ± 3.05	−0.891	0.383
Act	23.75 ± 4.69	23.75 ± 4.69	−0.230	0.820
FFMQ	131.50 ± 22.215	130.25 ± 12.38	0.170	0.488

SSA: somatic state anxiety. CSA: cognitive state anxiety. SSC: state of self-confidence.

### 3.2 CSAI-2 after mindfulness intervention

Table 4 indicates a decrease in the somatic anxiety of the mindfulness group mid- and post-intervention. Repeated measure ANOVA shows significant main effects for groups (*F* = 16.23, *p* < 0.001, and *η^2^*_p_ = 0.43), time (*F* = 5.05, *p* < 0.019, and *η^2^*_p_ = 0.19), and interaction (*F* = 7.60, *p* < 0.004, and *η^2^*_p_ = 0.257). The simple effects of groups are not significant at pre-intervention (*F* = 0.002, *p* = 0.965, and *η^2^*_p_ = 0.000) but significant at mid-intervention (*F* = 6.29, *p* = 0.019, and *η^2^*_p_ = 0.225) and post-intervention (*F* = 60.90, *p* = 0.001, and *η^2^*_p_ = 0.735).

**Table 4:** Means, standard deviations, and two-way ANOVA statistics for outcome measures.

	MAIC	Control	Effect	ANOVA		
Measures	M ± SD	M ± SD		*F*	*P*	η^2^_p_
SSA						
Pre-intervention	27.00 ± 10.18	26.83 ± 7.74	A	16.23	0.001	0.43
Intervening	19.33 ± 3.49	25.83 ± 8.18	T	5.05	0.019	0.19
Post-intervention	19.25 ± 5.81	24.91 ± 6.41	A × T	7.60	0.004	0.26
Csa						
Pre-intervention	27.00 ± 7.96	30.58 ± 6.34	A	15.70	0.001	0.42
Intervening	22.00 ± 10.46	30.16 ± 8.92	T	4.19	0.02	0.16
Post-intervention	15.41 ± 8.59	21.58 ± 7.69	A × T	5.24	0.01	0.19
SSC						
Pre-intervention	38.91 ± 8.65	35.33 ± 7.16	A	8.49	0.008	0.28
Intervening	39.25 ± 7.77	33.50 ± 4.90	T	0.49	0.62	0.02
Post-intervention	42.16 ± 8.89	34.33 ± 5.14	A × T	2.60	0.09	0.11
Csai-2						
Pre-intervention	92.91 ± 14.73	90.66 ± 14.02	A	5.22	0.03	0.99
Intervening	80.58 ± 11.57	89.50 ± 16.01	T	6.43	0.004	0.23
Post-intervention	70.41 ± 5.55	89.50 ± 14.08	A × T	5.20	0.009	0.19

M represents the average; SD represents the difference in samples; A represents the effect size of the group; T represents the effect size of time; A × T represents the interaction between the group and time; F represents the variance analysis; P represents significance; η^2^_p_ represents the effect size.

Multiple comparisons reveal that somatic anxiety levels decreased progressively from mid-test to post-test within the mindfulness group. All results indicate significance (*p* < 0.05). Somatic anxiety is reduced sequentially from the mid- and post-tests in the control group, indicating no significance (*p* > 0.05). Somatic anxiety decreases at pre-, mid-, and post-intervention, reaching significant levels. There is a significant difference between pre- and mid-tests (*p* < 0.024), mid- and post-tests (*p* < 0.004), and pre- and post-tests (*p* < 0.001) in the mindfulness group. No difference exists among the pre-, mid-, and post-tests in the control group.

Table 4 displays increased and unchanged SSC in the mindfulness group and control group during and after the intervention. Repeated measure ANOVA results indicate that groups have significant main effects (*F* = 8.49, *p* = 0.008, and *η^2^*_p_ = 0.28); however, those of time (*F* = 0.49, *p* = 0.62, and *η^2^*_p_ = 0.02) and interaction effects between time and groups (*F* = 2.60, *p* = 0.09, and *η^2^*_p_ = 0.11) are not significant. The test of groups’ simple effects shows that they are not significant at pre-intervention (*F* = 1.22, *p* = 0.28, and *η^2^*_p_ = 0.05.) but significant at mid-intervention (*F* = 4.70, *p* = 0.04, and *η^2^*_p_ *= 0*.*18*) and post-intervention (*F* = 11.97, *p* = 0.002, and *η^2^*_p_ = 0.35). The analysis of the simple effects of time reveals no significant differences in the mindfulness group (*F* = 0.38, *p* = 0.69, and *η^2^*_p_ = 0.04) and control group (*F* = 1.93, *p* = 0.17, and *η^2^*_p_ = 0.16).

The table demonstrates the decreased and unchanged CSA of the mindfulness group and the control group during and after the intervention. Repeated measures ANOVA indicates main effects are significant in groups (*F* = 15.70, *p* = 0.001, and *η^2^*_p_ = 0.42) and time (*F* = 4.19, *p* = 0.02, and *η^2^*_p_ = 0.16). However, they are not significant in the interaction between groups and time (*F* = 5.24, *p* = 0.01, and *η^2^*_p_ = 0.19). The simple effects of groups shows that they are not significant at the pre-intervention (*F* = 1.49, *p* = 0.24, and *η^2^*_p_ = 0.06) and mid-intervention (*F* = 4.23, *p* = 0.05, and *η^2^*_p_ = 0.16) but significant at the post-intervention (*F* = 48.88, *p* = 0.001, and *η^2^*_p_ = 0.69). The simple effects of time shows that they are significant in the mindfulness group (*F* = 8.37, *p* = 0.002, and *η^2^*_p_ = 0.45) but not significant in the control group (*F* = 0.08, *p* = 0.92, and *η^2^*_p_ = 0.01). Multiple comparisons indicate pre-, mid-, and post-tests sequentially decrease CSA due to mindfulness intervention. There are significant differences in CSA between pre- and post-tests (*p* = 0.001) as well as mid- and post-tests CSA (*p* = 0.017) in the mindfulness group. However, no significant difference exists in the CSA of pre-, mid-, and post-tests in the control group.

There is decreased and unchanged CSAI-2 in the mindfulness group and control group during and after the intervention. Repeated measures ANOVA results show significant main effects exist in groups (*F* = 5.22, *p* = 0.03, and *η^2^*_p_ = 0.99) and time (*F* = 6.43, *p* = 0.004, and *η^2^*_p_ = 0.23). However, they are not significant in the interaction between time and groups (*F* = 5.20, *p = 0*.009, and *η^2^*_p_ = 0.19). The simple effects of groups show that they are not significant in the pre- and mid-tests but significant in the post-test (*F* = 19.08, *p* = 0.001, and *η^2^*_p_ = 0.46). The simple effects of time show they are significant time due to the mindfulness intervention (*F* = 8.92, *p* = 0.002, and *η^2^*_p_ = 0.46), but not significant under control conditions (*F* = 0.03*, p* = 0.97, and *η^2^*_p_ = 0.00). Multiple comparisons reveal that the CSAI-2 values of pre-, mid-, and post-tests are sequentially lower due to the mindfulness training. The CSAI-2 values have significant differences between pre- and mid-tests (*p* = 0.014), mid- and post-tests (*p* = 0.023), and pre- and post-tests (*p* = 0.001) in the mindfulness group. However, no significant differences exist in the control group.

### 3.3 FFMQ after mindfulness intervention

Table 5 shows mean scores on all aspects of mindfulness increase over time, but there are slight differences in scores across aspects. This trend is supported by the within-subjects variance. The observing dimension is statistically significant considering the main effects of time (*F* = 3.37*, p* = 0.04, and *η^2^* = 0.13) and groups (*F* = 14.33, *p* = 0.001, and *η^2^*_p_ = 0.39). The non-reacting to inner experience dimension is significant considering the main effect of the group (*F* = 4.26, *p* = 0.05, and *η^2^*_p_ *= 0*.16) and interaction effects of group and time (*F* = 6.60, *p* = 0.003, and *η^2^*_p_ = 0.23). Multiple comparisons show that scores on observing, describing, and non-reacting to inner experience dimensions are statistically significant during and after intervention (*p* < 0.05).

**Table 5:** Means, standard deviations, and two-way ANOVA statistics for outcome measures.

	MAIC	Control	Effect	ANOVA		
Measures	M ± SD	M ± SD		*F*	*P*	*η^2^*_p_
Observe						
Pre-intervention	29.833 ± 6.464	28.583 ± 7.216	A	14.33	0.001	0.39
Intervening	32.500 ± 3.233	26.750 ± 5.276	T	3.37	0.04	0.13
Post-intervention	37.416 ± 5.45	28.500 ± 5.402	A × T	2.96	0.06	0.12
Describe						
Pre-intervention	27.083 ± 6.694	26.083 ± 4.378	A	3.90	0.06	0.15
Intervening	28.000 ± 4.327	26.166 ± 4.427	T	1.81	0.17	0.08
Post-intervention	31.500 ± 5.616	26.500 ± 3.919	A × T	1.24	0.30	0.05
Non-judge						
Pre-intervention	27.583 ± 6.556	26.583 ± 4.316	A	0.92	0.35	0.04
Intervening	28.583 ± 3.117	28.416 ± 4.851	T	1.21	0.31	0.05
Post-intervention	30.250 ± 4.287	27.250 ± 5.154	A × T	0.79	0.46	0.03
Non-react						
Pre-intervention	23.666 ± 5.348	25.250 ± 3.048	A	4.26	0.05	0.16
Intervening	26.083 ± 6.200	23.416 ± 3.824	T	2.14	0.13	0.09
Post-intervention	30.333 ± 5.898	23.166 ± 2.790	A × T	6.60	0.003	0.23
Act						
Pre-intervention	23.333 ± 4.163	23.750 ± 4.692	A	1.01	0.33	0.04
Intervening	22.500 ± 4.078	25.666 ± 6.169	T	2.67	0.08	0.11
Post-intervention	20.583 ± 5.664	21.083 ± 6.999	A × T	0.54	0.59	0.02
Ffmq						
Pre-intervention	127.416 ± 21.873	126.083 ± 13.500	A	5.58	0.027	0.20
Intervening	132.083 ± 12.915	133.833 ± 19.237	T	1.60	0.21	0.06
Post-intervention	144.833 ± 15.330	126.500 ± 15.733	A × T	3.87	0.028	0.15

M represents the average; SD represents the difference in samples; A represents the effect size of the group; T represents the effect size of time; A × T represents the interaction between the group and time; F represents the variance analysis; P represents significance; η^2^_p_ represents the effect size.

The FFMQ scores are improved significantly with mindfulness intervention. Repeated measures ANOVA shows significant main effects in groups (*F* = 5.58, *p* = 0.027, and *η^2^*_p_ = 0.20) and the interaction between time and groups (*F* = 3.87, *p* = 0.028, and *η^2^*_p_ = 0.15). However, they are not significant in time (*F* = 1.60, *p* = 0.21, and *η^2^*_p_ = 0.06). The simple effects of groups show they are not significant before intervention (*F* = 0.02, *p* = 0.87, and *η^2^*_p_ = 0.001) and during intervention (*F* = 1.50, *p* = 0.23, and *η^2^*_p_ = 0.06), but significant after intervention (*F =* 13.37, *p* = 0.001, and *η^2^*_p_ = 0.38). The simple effects of time show that they are significant in the mindfulness group (*F* = 5.62, *p* = 0.011, and *η^2^*_p_ = 0.35), but not significant in the control group (*F* = 0.32, *p* = 0.73, and *η^2^*_p_ = 0.02). Multiple comparisons indicate that the FFMQ values of pre-, mid-, and post-tests increase sequentially in the mindfulness group. The describing dimension values are significantly different between the pre- and post-tests (*p* = 0.003) as well as the mid- and post-tests (*p* = 0.040). No significant differences exist between observed dimensions (*p* = 0.336) in pre- and mid-tests. The dimensions are not significantly different in the control group in pre-, mid-, and post-tests.

## 4 Discussion

The effects of 7-week MAIC on the competitive state anxiety of sprinters were examined. Seven-week MAIC improved mindfulness and SSC and reduced SSA and CSA.

Mindfulness training was designed to help sprinters develop mindfulness skills and improve athletic performance. Athletes’ level of mindfulness was measured using the FFMQ before, during, and after the intervention. The FFMQ could capture changes in mindfulness across experimental phases. The observed dimensions increased at all three-time points and were statistically significant. The non-reacting to the inner experience dimension was significant considering the interaction effects of time and group. Scores on observing, describing, and non-reacting to inner experience dimensions increased at all three-time points. However, the increases were statistically significant before and after the intervention. These results were consistent with published literature, suggesting that observing and non-reacting to inner experience dimensions were stronger inverse predictors of emotional symptoms, including fear and anxiety (Medvedev et al., 2018).

Competitive state anxiety was significantly reduced due to MAIC intervention, suggesting that mindfulness intervention was a key to clinical improvement. Fairly strong evidence suggested that mindfulness programs [e.g., mindfulness-based stress reduction therapy (Kabat-Zinn, 2013)] reduced the competitive anxiety level in athletes (Gan et al., 2024). However, flow states (Liu, 2023) have shown promising results for psychological health, suggesting the utility of the mindfulness intervention in the study.

Previous research has shown that mindfulness training can increase athletes’ mindfulness levels and promote athletes’ performance (Zhang et al., 2016). It is confirmed by comparing the results of the mindfulness and control groups. However, only the observed dimensions and the FFMQ have the main effect of time. The likely explanation is that athletes’ engagement in mindfulness exercises is limited, and they have not fully integrated these practices into their routines.

Results from recent studies and meta-analyses suggest that the mindfulness intervention can alleviate athletes’ anxiety levels (Deck et al., 2022). Additionally, it provides a strong evidence base for applying psychological intervention to reduce athletes’ competitive state anxiety (Ong and Chua, 2021). Athletes in the mindfulness group decrease SSA and CSA and increase SSC. Importantly, this positive effect is immediately visible in the training, demonstrating the relative immediacy of the mindfulness training. These results are similar to those of imagery (Fekih et al., 2021) and self-talk training (Walter et al., 2019), which are commonly used in sports psychology training. The process integration hypothesis proposed by Wang suggests that athlete choking arises because of personality factors, performance expectations, and spectators (Jing et al., 2018). Mindfulness practice prompts athletes to cultivate an open and non-judgmental attitude toward their present thoughts and emotions. It reduces self-critical thinking, which can be distressing (Gardner and Moore, 2017). Consequently, mindfulness training can help improve athletes’ psychological conditions during competition.

## 5 Conclusion

Mindfulness training has been systematically applied to sports as a psychological intervention. Mindfulness training was innovatively applied to measure the changes in the competition anxiety of sprinters under mindfulness intervention. Mindfulness intervention could significantly improve the mindfulness level of sprinters and reduce the anxiety of sprinters in competition. Mindfulness training after regular training sessions increased athletes’ self-confidence and reduced physical state anxiety and cognitive state. Specifically, enhanced self-confidence improved the sports performance of athletes because the subjects in the study have achieved good results in national track and field competitions. The psychological problems of sprinters have become a common concern in the sports training theory and practice with the rapidly growing international competitiveness of China’s sprint program. Athletes were recommended to arrange mindfulness training rationally according to their convenience to obtain the ideal psychological state and sports performance in daily training or competitions.

## 6 Limitations

Limitation 1: Small sample sizes may reduce the effectiveness of the study due to the limited number of sprinters who meet our inclusion and exclusion criteria. Limitation 2: The control group could not receive conventional psychological counseling due to the researchers’ limited professionalism and manpower. Therefore, the results of the study should be interpreted cautiously.

## Data Availability

The original contributions presented in the study are included in the article/Supplementary material, further inquiries can be directed to the corresponding author.
